# Adherence of iron and folic acid supplementation and determinants among pregnant women in Ethiopia: a systematic review and meta-analysis

**DOI:** 10.1186/s12978-019-0848-9

**Published:** 2019-12-21

**Authors:** Melaku Desta, Bekalu Kassie, Habtamu Chanie, Henok Mulugeta, Tadesse Yirga, Habtamu Temesgen, Cheru Tesema Leshargie, Yoseph Merkeb

**Affiliations:** 1 Department of Midwifery, College of Health Science, Debre Markos University, PO. Box: 269, Debre Markos, Ethiopia; 2 Department of Nursing, College of Health Science, Debre Markos University, Debre Markos, Ethiopia; 3 Department of Nutrition and Food Science, College of Health Science, Debre Markos University, Debre Markos, Ethiopia; 4 Department of Public Health, College of Health Science, Debre Markos University, Debre Markos, Ethiopia; 5 Department of Biomedical Science, College of Health Science, Debre Markos University, Debre Markos, Ethiopia

**Keywords:** Adherence, Determinants, Iron-folic acid, Meta-analysis, Ethiopia

## Abstract

**Background:**

Iron and folic acid deficiency anaemia are one of the global public health challenges that pose 1.45% of all disability-adjusted life-years. It is recognized as a cause for an unacceptably high proportion of maternal and perinatal morbidity and mortality. Adherence to iron and folic acid supplementation during the antenatal period is paramount to reduce anaemia and its associated morbidities. Although several studies have been conducted across the country, their reports were inconsistent and inconclusive for intervention. Therefore, this systematic review and meta-analysis were aimed to estimate the pooled national level adherence to iron and folic acid supplementation and its determinants among pregnant women in Ethiopia.

**Methods:**

This systematic review and meta-analysis were pursued the Preferred Reporting Items for Systematic Reviews and Meta-Analyses (PRISMA) 2009 guideline. An extensive search of databases including, PubMed, Google Scholar, and African Journals Online were conducted to access articles. The Newcastle- Ottawa quality assessment tool was used to assess the quality of each study and meta-analysis was conducted using a random-effects model. I^2^ test and Egger’s test were used to assess the heterogeneity and publication bias respectively. The meta-analysis of estimating national level adherence were done using STATA version 11 with 95% CI.

**Results:**

Twenty studies with a total of 16,818 pregnant women were included in this meta-analysis. The pooled national level iron and folic acid supplementation’s adherence were 46.15% (95%CI:34.75,57.55). The highest adherence was observed in Addis Abeba, 60% (95%CI: 55.93, 64.07) followed by Tigray, 58.9% (95% CI: 33.86, 84.03). Women who received supplemental information [OR = 2.34, 95%CI: 1.05, 5.24], who had good knowledge [OR = 2.2, 95%CI: 1.05, 5.24], began the ANC visit before 16 weeks [OR = 2.41, 95%CI: 1.76, 3.29], and had ≥4 ANC visits [OR = 2.59, 95% CI: 1.09, 6.15] were more likely adhere to the supplementation. Fear of side effects (46.4, 95% CI: 30.9 61.8) and forgetfulness (30.7, 95% CI: 17.6, 43.8) were the major barriers of adherence of the supplementations.

**Conclusions:**

More than four of nine pregnant women have adhered to the iron and folic acid supplementation**.** This meta-analysis revealed that receiving supplemental counselling, knowledge of the supplement; early registration and frequent ANC visit were significantly associated with the adherence of the iron and folic acid supplementation. Therefore, provision of strengthened supplemental counselling service, antenatal care services, and improving the knowledge of the supplementation is a crucial strategy to increase the adherence among pregnant women in Ethiopia. Besides, addressing the barriers of the adherence of the supplement mainly counseling or managing of side effects and reducing of forgetfulness to take the tablet through getting family support or male involvement during visit is mandatory.

## Plain English summary

Iron and folic acid deficiency anaemia is a global public health problem that causes high maternal morbidity and mortality. Proper provision and utilization of iron and folic supplementations for pregnant women prevent the threat of maternal health and perinatal outcomes. Previous studies on the adherence of iron and folic acid supplementation among pregnant women were inconsistent across the country. Therefore, this systematic review and meta-analysis estimate the pooled adherence of iron and folic acid supplementation and determinants among pregnant women in Ethiopia. The systematic review and meta-analysis were followed the Preferred Reporting Items for Systematic Reviews and meta-analyses guideline and PubMed, Cochrane Library, Google Scholar, and African Journals Online databases were used for searching.

Two authors were systematically extracted the data using predetermined inclusion criteria to Microsoft excel and meta-analysis was done using STATA version 11 software. I^2^ test was used to assess the heterogeneity and Eggers test for publication bias of included studies. Moreover, a random-effects meta-analysis model was computed to estimate the pooled adherence of IFA supplementation. Subgroup analysis was done based on the region of study. The pooled national level of adherence towards iron and folic acid supplementation during their antenatal care (ANC) visit based on the WHO recommendation among pregnant women were 46.5%. The adherence of IFA supplementation was significantly associated with receiving counselling, knowledge of the supplement, early registration and frequency of ANC visit. Besides, fear of side-effects and forgetfulness were the major barriers of adherence. Therefore, addressing those factors is paramount to improve the adherence to iron and folic acid supplementation.

## Introduction

Globally, about 38.2% of pregnant mothers are anaemic. Of these, almost two-thirds are in developing countries [1]. In Africa, prenatal anemia was detected in 48.7% of mothers [2]. Iron and folic acid (IFA) deficiency anaemia is one of the causes for 1.45% of all the disability-adjusted life years (DALYs) [3], and the leading cause of malnutrition and more than half of anemia which resulting in adverse maternal and perinatal outcomes throughout the life-course [4–6]. Anaemia increase risk of mortality, morbidity, postpartum hemorrhage, poor birth outcomes, preterm births and low birth weight (LBW) [7].

World Health Organization (WHO) and Ethiopian Government recommended initiating the daily Iron and Folic Acid (IFA) supplementation during pregnancy as early as possible as a part of ANC programs for positive pregnancy outcome [8–10]. A meta-analysis revealed that adherence of IFA supplementation is means of reduction of anemia at term by 69% and LBW by 20% [11], and it reduces risk of early neonatal and under-five mortality by 45 and 55% in Nepal [12], neonatal death by 34% in Pakistan [13] and the risk of stunting in children by 23% [14, 15]. Similarly, other studies show that prenatal IFA supplementation is recommended for protection against maternal and neonatal death [7, 10, 16, 17], anaemia and LBW [18–20], symptoms of preeclampsia [21] and improves overall pregnancy outcomes [22, 23].

The adherence of IFA supplementation vary across the country [18, 24–27] and several different determinants are associated with the adherence of the supplementation [28–31]. Despite improvements in maternal and child health programs in the last 2 decades [32, 33], the burden of anaemia remains a common problem among pregnant women in Ethiopia, 24% [6, 34], and adherence of IFA supplement is inconsistent. The IFA status of pregnant women has not permanently and universally improved through addressing of information and reduction of barriers of adherence of IFA supplementation. For this, estimating the national level of adherence to iron and folic acid supplementation is paramount. Hence, the studies are inconsistent and inconclusive across the country for policymakers or evidence-based interventions. Therefore, this systematic review and meta-analysis were aimed to estimate the pooled adherence of IFA supplementation and its determinants among pregnant women in Ethiopia.

## Methods

### Systematic review registration and reporting of findings

The protocol has been registered on an International Prospective Register of Systematic Review (PROSPERO), University of York Center for Reviews and Dissemination (https://www.crd.york.ac.uk/), registration number CRD42018106313 and the findings of the review was reported in following the recommendation of the Preferred Reporting Items for Systematic Review and Meta-Analysis (PRISMA-P) 2009 statement guideline [35].

### Study design and search strategy

The systematic review and meta-analysis were designed to estimate the adherence of IFA supplementation and its determinants among pregnant women in Ethiopia. The major international databases such as PubMed, Cochrane Library, Google Scholar, and African Journals Online databases were searched from January 15 to March 25/2019 for all published studies; Google hand searching was also performed for unpublished studies. Besides, the search of the reference list of already identified articles was done to retrieve additional articles. All studies those were published from 2000 to March 09/2019 and unpublished study mainly, the University repositories Addis Abeba and Haramya University were retrieved to assess for the eligibility of inclusion in this review and critical appraisal.

The PECO (Population, Exposure, Comparison and Outcomes) search has used this review.

Population**:** Pregnant women who have received IFA supplementation during their ANC visit.

Exposure: determinants of adherence of IFA supplementation (knowledge-related factors such as receiving information on the supplementation, knowledge of the supplementation and Knowledge of anemia; Antenatal related factors (timing of ANC visit and frequency of ANC visit) and history of anemia.

Comparison: The reported reference groups for each determinant factor in each respective study such as, adherence among pregnant women who have received information on the IFA supplementation versus those who haven’t receive the information, and adherence among women who started ANC visit before 16 weeks versus their counterparts.

Outcome: the adherence of IFA supplementation among pregnant women.

The Electronic databases were searched with keyword searching and using the medical subject heading [MeSH] terms for each selected PECO component. The keyword searching includes adherence, compliance, “Iron-folic acid, Iron and folic acid, pregnant and Ethiopia. The Boolean operators “OR” and “AND” were used to combine the searching terms.

### Eligibility criteria and study selection

This review included studies that were reported the adherence of IFA supplementation or the determinants of IFA supplementation or the barriers of adherence among pregnant women in Ethiopia. All studies conducted at the community or health institution level, published and unpublished in the English language from 2000 to March 09/2019 were included. Whereas, studies conducted within the study populations other than pregnant women, case reports, surveillance data (Demographic health survey), conference abstracts, and articles without full access were excluded from the review. In the screening phase, two reviewers (MD and BK) assessed the articles independently for inclusion through a title, abstract and full review. Any disagreement was solved by a consensus with the two reviewers and if necessary with the third reviewer (HC). In the second phase of screening, those potentially eligible studies were undergoing full-text review to determine if they satisfy the predetermined inclusion criteria and assessed for duplicated records. When duplicate data were encountered, only the full-text article published was retained.

#### Outcome of interest

The primary outcome of this review was the adherence of IFA supplementation among pregnant women. Adherence of IFA supplementation was defined by WHO, a woman who had taken iron folate supplements ≥90 days or 4 days per week during the pregnancy period considered as adhered to iron-folate supplementation [1].

The secondary outcomes were: the determinants of IFA supplementation adherence such receiving information on the IFA supplementation, knowledge of IFA supplementation and anemia, history of anemia, the timing of ANC visit and frequency of ANC visit (having 4 and above visit and below 4 visits) and barriers of adherence (forget fullness, fear of side effects, too many pills and increase the size of the baby).

#### Quality assessment and data extraction

The Newcastle-Ottawa Scale quality assessment tool for the cross-sectional studies was used for assessing the quality of included studies based on the three components [36]. The principal component of the tool graded from five stars and mainly emphasized on the methodological quality of each primary study. The other component of the tool graded from two stars and mainly concerns about the comparability of each study and the last component of the tool graded from three stars and used to assess the outcomes and statistical analysis of each original study. Then, the two reviewers (MD and BK) independently assessed or extracted the articles for overall quality and or inclusion in the review using a standardized data extraction format. The data extraction format included primary author, publication year, and region of the study, sample size, prevalence, determinants and barriers of adherence.

#### Publication bias and, Statistical analysis

The publication bias was assessed using Egger’s tests. A *p*-value of less than 0.05 was used to declare a statistically significant presence of publication bias. I^2^ test statistics were used to investigate the heterogeneity across the included studies. The I^2^ test statistics of 25, 50 and 75% was declared as low, moderate and high heterogeneity respectively and a p-value less than 0.05 was used to declare significant heterogeneity. For the test result with the presence of heterogeneity, a random effect model was used as a method of analysis. Data were extracted in Microsoft Excel and then exported to STATA version 11 for further analysis. Forest plot was used to present the combined estimate with 95% confidence interval (CI) of the meta-analysis. Subgroup analysis was conducted by regions of the country and barriers of adherence. Besides, a meta-regression model was done based on sample size and year of publication to identify the sources of random variations among included studies. The effect of selected determinant variables which include; receiving information on the IFA supplementation, knowledge of IFA, knowledge of anemia, history of anemia, the timing of ANC visit, frequency of ANC visit and four barriers of adherence of IFA supplement were analyzed using separate categories of meta-analysis. The findings of the meta-analysis were presented using forest plot and Odds Ratio (OR) with its 95% CI.

## Results

### Study identification

**Fig. 1:**
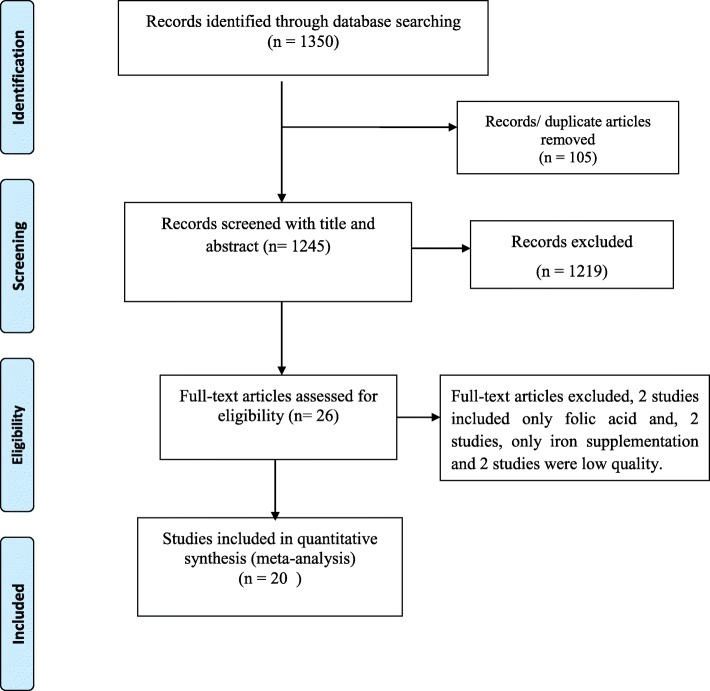
PRISMA flow diagram of adherence of IFA supplementation in Ethiopia

This systematic review and meta-analysis included published and unpublished studies on the adherence of IFA supplementation among pregnant women in Ethiopia. The review found a total of 1350 articles, 1345 published articles and 5 unpublished articles. From those, 105 duplicated records were removed and 1219 articles were excluded through screening of the title and abstracts due to irrelevance and location outside of Ethiopia. After that, a total of 26 full-text papers were assessed for eligibility based on the inclusion and exclusion criteria. Finally, 20 studies were included for the final meta-analysis. Hence, six studies were excluded due to different reseasons; 2 articles were excluded because of the outcome of interest was not reported, two articles report only iron supplementation and the remaining 2 studies were reported only on the folic acid supplementation alone without iron (Fig. 1).

### Characteristics of the included studies

All included studies were cross-sectional comprising of 16,818 pregnant women to estimate the pooled adherence of IFA supplementation. The study conducted with a maximum sample size of 7764 of participants done at National level [37] and the minimum sample, 241 were conducted at Debre Tabor Hospital, Amhara region [38]. The studies were conducted between 2015 and 2019 across the country, most studies conducted in the five regions. Of those studies, 7 were from the Amhara region, 5 from Southern Nations, Nationalities and Peoples Representative (SNNPR), and 3 from Tigray. The quality of most studies was high (Table 1). Moreover, regarding the publication of the articles, 19 articles were published and only one study was unpublished [55].

**Table 1:** Characteristics of the included studies in the meta-analysis, Ethiopia

Author	Year	Region	Adherence	Sample	Response rate	Quality
Taye et al. [26]	2015	Amhara	20.4	628	99%	Moderate
Birhanu TM et al. [39]	2018	Amhara	55.3	418	100%	High
Gebremariam AD et al. [38]	2019	Amhara	44	241	92%	High
Gebreamlak B et al. [40]	2017	Addis Abeba	60	557	88.9%	High
Gebremedhin S et al. [41]	2014	Tigray	74.9	1563	97.1%	High
Haile MT et al. [42]	2017	Oromia	18	405	95.9	Moderate
Nigusie W [43]	2018	Oromia	59.8	317	93.6%	High
Gebre A et al. [44]	2017	Afar	22.9	450	98.4%	Moderate
Gebre A et al. [45]	2015	Tigray	37.2	714	100	Moderate
Getachew M et al. [46]	2018	Tigray	64.7	320	100	High
Sadore A et al. [47]	2015	SNNPR	39	303	97.6%	High
Shewasinad &Negash [48]	2017	SNNPR	70.6	462	93%	Moderate
Jikamo and Samuel [49]	2018	SNNPR	69.6	365	86.6%	High
Molla T et al. [50]	2019	Amhara	52.9	348	100%	High
Derso HD et al. [51]	2018	Amhara	28.7	418	100%	High
Boti N et al. [52]	2018	SNNPR	51.4	317	100	High
Demis A et al. [53]	2018	Amhara	43.1	422	100	High
Kassa ZY et al. [54]	2019	SNNPR	38.3	422	95.3%	High
Yadeta and Berhanu [55]	2016	Amhara	55.5	384	100	Moderate
Haile D et al. [37]	2017	National	17.1	7764	95%	Moderate

#### Adherence of IFA supplementation

**Fig. 2:**
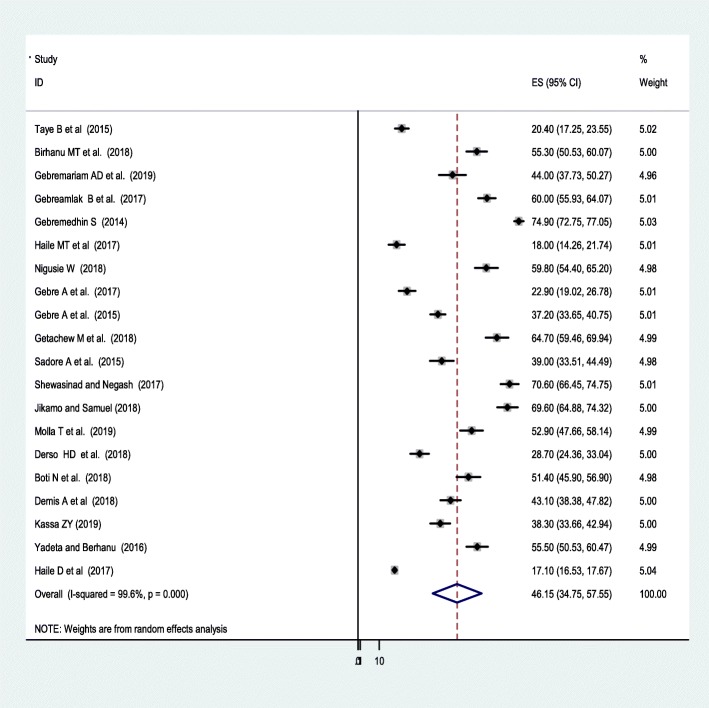
The pooled adherence of IFA supplementation among pregnant in Ethiopia

The lowest adherence rate of IFA supplement was 17.1% observed in a study conducted at the national level [37] and the highest, 74.5% was observed in a study conducted by the Tigray region [41]. The I^2^ (variation in ES attributable to heterogeneity) test result showed that there was high heterogeneity with I^2^ = 97.7% at the *p*-value = ≤ 0.05. For this, the pooled national level of adherence of IFA supplementation among pregnant women was 46.15% (95% CI: 34.75, 57.55) based on the random effect analysis (Fig. 2).

A univariate meta-regression revealed that there is no significant heterogeneity due year of publication (*p* = 0.61) and sample size (*p* = 0.42), and the Duval and filled analysis was conducted due to there was a publication bias and to fill with the unpublished studies.

A subgroup analysis by region was computed to compare the adherence of IFA supplementation across regions of the country. Accordingly, the highest adherence to IFA supplementation was observed in Addis Abeba, 60% (95%CI: 55.93, 64.07) followed by Tigray region, 58.9 (95%CI: 33.86, 84.03). Whereas, the lowest adherence,19.67%(95%CI:14.02,25.32) to the supplementation was observed among other regions, in the Afar region **(**Table 2). The Egger’s test of this meta-analysis revealed that there was a significant publication bias (*P*-value 0.025).

**Table 2:** Subgroup analysis of IFA supplementation adherence by region

Region	No of included studios	Prevalence (95%CI)	*P*-value	I^2^
Amhara	7	42.78 (31.04, 54.51)	< 0.0001	97.8
SNNPR	5	53.82 (39.5, 68.14)	< 0.0001	97.7
Tigray	3	58.94 (33.86, 86.03)	< 0.0001	99.4
Oromia	2	38.85 (2.11, 79.82)	< 0.0001	99.4
Others	2	19.67 (14.02, 25.32)	0.004	88.1
Addis Abeba	1	60 (55.93, 64.07)		

Others: Afar and national level study

### Determinants of Iron folic acid supplementation adherence

#### Knowledge related factors and history of anemia

**Fig. 3:**
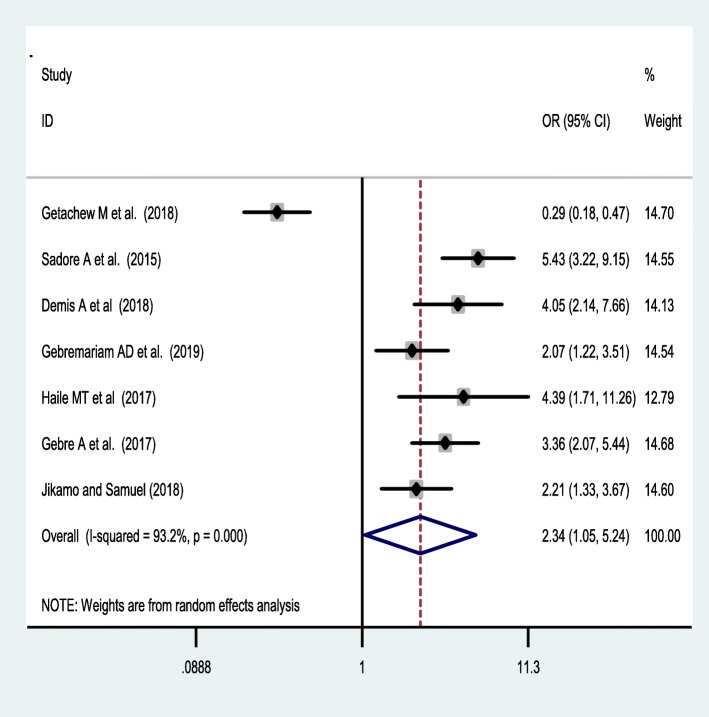
forest plot on the effect of receiving information on the supplement and its adherence in Ethiopia

**Fig. 4:**
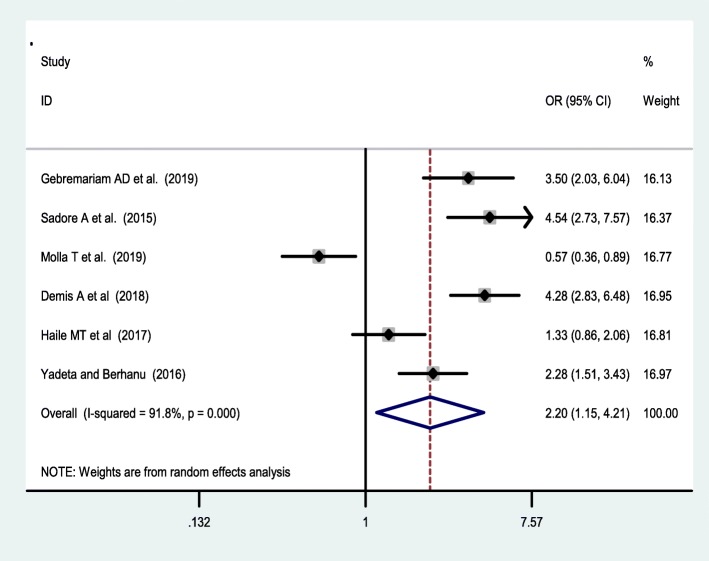
Association of Knowledge of IFA supplementation and IFAS adherence in Ethiopia

**Fig. 5:**
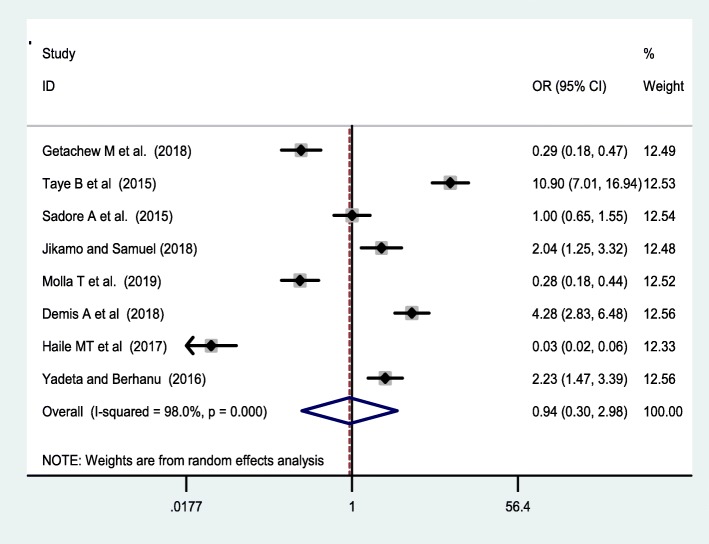
forest plot of on the association of knowledge of anemia with IFA adherence

**Fig. 6:**
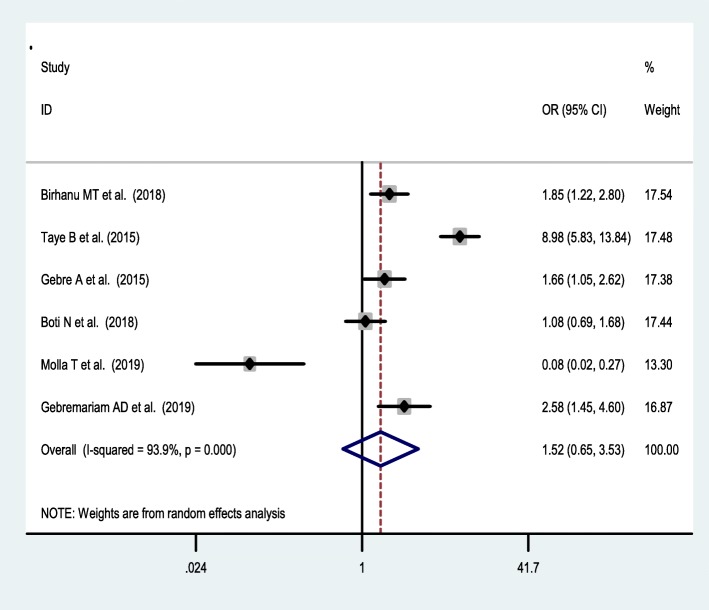
forest plot of association of history of anemia and adherence of IFAS in Ethiopia

Seven studies [38, 42, 44, 46, 47, 49, 53] were included to assess the effect of receiving information towards IFA supplementation adherence. Pregnant women who have received counselling or information on the IFA supplementation were 2.34 [OR = 2.34,95%CI:1.05,5.24] times more likely to have good adherence of the supplementation. The heterogeneity test showed that there was statistically significant evidence of heterogeneity, I^2^ = 93.2% and *P* = 0.001 **(**Fig. 3**).** Egger’s test showed a non-significant publication bias. Besides, six studies [38, 42, 47, 50, 53, 55] were considered to assess the association between knowledge of IFA supplementation and adherence IFA supplementation. Thus, women who have a good knowledge regarding the IFA supplement were two [OR = 2.2, 95%CI: 1.05,5.24] times more likely adhere to the IFA supplementation compared with their counterparts. Based on the I^2^ test (I^2^ = 91.8%) and a *p*-value of < 0.05, we observed the presence of significant heterogeneity across (Fig. 4), and there was a publication bias based on the egger’s test. Moreover, 8 studies [26, 42, 46, 47, 49, 50, 53, 55] were included to assess the association of knowledge of anaemia and 6 studies were included on the association of history of anaemia [26, 38, 39, 45, 50, 52] with the adherence of IFA supplementation. However, neither knowledge of anemia nor history of anemia were associated with the adherence of the IFA supplementation. Random effect model was used for both factors due to the presence of a significant heterogeneity with I^2^ = 98% or 93% at *p*-value < 0.05 **(**Figs. 5 and 6**).**

### Antenatal care-related factors

#### Timing of antenatal care visit

**Fig. 7:**
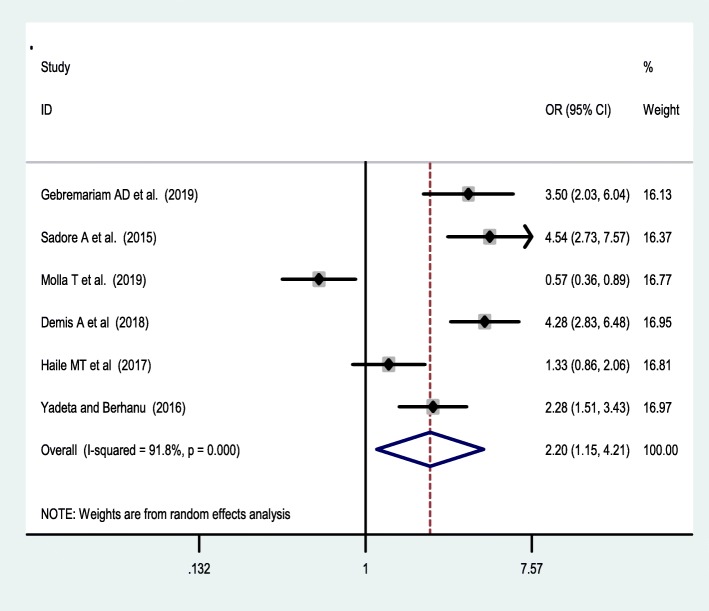
forest plot of effect of early registration on adherence of IFA supplementation in Ethiopia

Based on the meta-analysis of six articles [38, 39, 45, 52, 53, 55], time of ANC registration significantly associated with the adherence to IFA supplementations, which pregnant women who have started their ANC visit before 16 weeks were 2.41 [OR = 2.41, 95%CI:1.76,3.29] times more likely adhered to the IFA supplementation based on the recommendations than those started their visit later than 16 weeks **(**Fig. 7**).** The Egger’s test showed no significant publication bias.

### Frequency of ANC visit

**Fig. 8:**
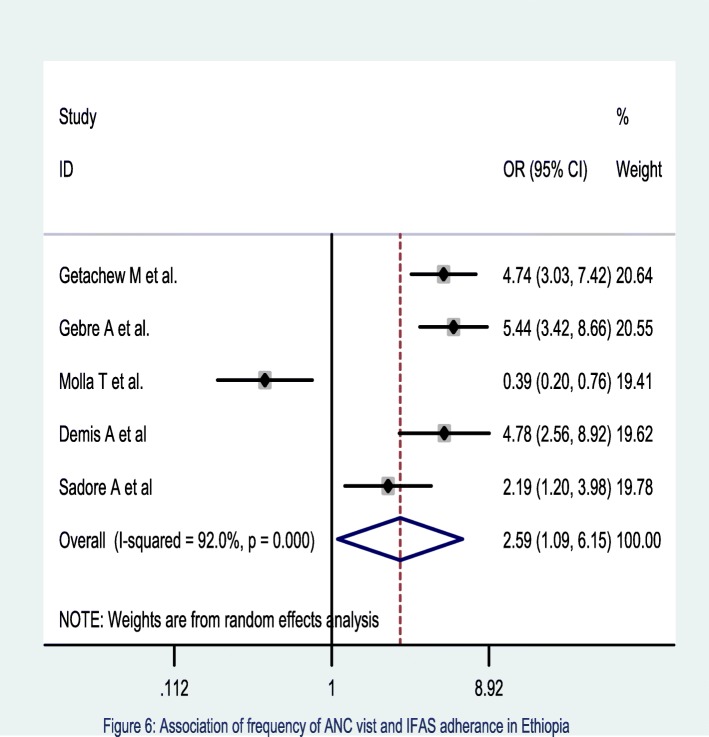
Association of Frequency of ANC visit and IFAS adherence in Ethiopia

The meta-analysis of five studies [45–47, 50, 53] also showed that those pregnant women who had four and above ANC visits were 2.59 times [OR = 2.59, 95% CI: 1.09, 6.15] more likely to adhere the recommended supplementation of IFA compared to their counterparts. The heterogeneity test showed a statistical evidence of variation, I^2^ = 92% at *p* = < 0.001(Fig. 8). Egger’s test showed no statistical evidence of publication bias.

### Barriers of adherence to iron-folic acid supplementation

The commonest reasons for non-adherence of IFA supplementations were associated with fear of side effects, 46.4% (95%CI: 30.92, 61.88) and forget fullness 30.74% (95%CI: (17.62, 43.89) (Table 3).

**Table 3:** Barriers of adherence of IFA supplementation in Ethiopia: meta-analysis

Barriers	No of included studies	Percentage (95%CI)	*P*-value	I^2^
Forgetfulness	7	30.75 (17.62, 43.89)	< 0.0001	99.1
Fear of side effects	7	46.40 (30.92, 61.88)	< 0.0001	99.1
Too many tablet	3	26.71 (−1.36, 54.78)	0.062	99.5
Affects size of baby	3	30.05 (26.27, 35.87)	< 0.0001	72.2

## Discussion

Adherence of IFA supplementation plays a major role in the prevention and treatment of iron deficiency anaemia particularly among pregnant women whose iron demand increasing due to fetal and maternal requirement [11]. This systematic review and meta-analysis found that the pooled national level prevalence of IFA supplementation adherence among pregnant women during their ANC visit based on the WHO recommendation was 46.5% (95% CI: 34.75, 57.55) [56]. The current adherence of IFA supplementation estimate was comparable with findings reported from the Netherlands, 51% [57] and Malawi, 37% [58]. On the other hand, this finding was higher compared with the Ethiopian Demographic Health Survey (DHS) report of 2016 [6],5%, New Zealand national cohort,8% [59], India,24% [60] and of 22 low-income countries [8], 8% of pregnant women have adhered to the IFA supplementation. This discrepancy might be explained due to the intermittent nature of data collection of DHS data (once every 5 years), absence of supply chain information and might be women’s potential of recall bias, might underestimate current situation of the country. Moreover, the variation in the study period might also the possible reason for the discrepancy as; all studies included in this meta-analysis are recent mainly 16 studies were published after 2017. Also, the improvement made on maternal health-seeking behaviour and ANC visit might be the reason to increase the level of adherence in this setting. This estimate was also lower compared with the reports in Vietnam, 82% [9] and Niger,68% [61]. The observed variation might be due to the differences in socio-demographic characteristics and knowledge of IFA supplementation [62].

The subgroup analysis revealed that the pooled level of IFA supplementation adherence was varied across the regions. The highest estimates of IFA supplementation adherence were reported from Addis Ababa and Tigray region. This might since due to higher maternal health care service utilization mainly ANC visits in the region attribute to the increment in the adherence of IFA supplementation among these regions. Hence, a recent national-level study in Ethiopia from the DHS data supported that the highest utilization of ANC visit was spatially clustered in Addis Ababa (94%) and Tigray regions (65%) respectively with an increasing trend than other regions [63], which result in improving the adherence of IFA supplementation. Beyond this, socio-demographic characteristics, the lifestyle activities in the Addis Ababa region are associated with socioeconomic differences that might also attribute the difference in the adherence of IFA supplementation. This implies that other regions should commit to improve maternal healthcare service utilization and might sustainably increase adherence.

In this meta-analysis, pregnant women who got information on the IFA supplementation from health facility was had a greater chance of adhering to the recommended IFA supplementation than those who did not get any supplemental information during their ANC visit. This is supported by other studies done in India [60], Bangladesh [64], South Africa [65], Zimbabwe [28]. The possible explanation could be because of pregnant women who got information regarding IFA supplementation by healthcare provider might be more likely share the benefit of the supplement during counselling, become more knowledgeable about the supplement [50] and enhancing the awareness of mothers can substantially improve the adherence of the supplementation. Thus, implies that improving the way of providing nutrition-sensitive information in a cultural context and appropriate understandable manner is important.

Accordingly, this meta-analysis also revealed that women who have good knowledge of the IFA supplementation had greater odds of adhered to the recommended IFA supplementation than those mothers who had poor knowledge. The finding is supported by a study done in Indonesia [24], South Africa [65], and Kenya [66]. The possible explanation for this might be due to the fact that mothers who have good knowledge of the supplementation might attain a higher level of education, might be more likely to get information regarding Iron-folate requirement and understood educational messages delivered through different media outlets. Beyond this, knowledgeable women might be more concerned about their health and pregnancy outcome and more likely to utilize maternal healthcare service [67], such as had higher number of ANC visit and early initiation of ANC visit [68], leads better exposure for about IFA supplement, which further improves the adherence of the IFA supplementation.

Furthermore, this meta-analysis showed that pregnant women with timely registration of ANC visit (before 16 weeks) and receiving the recommended number of ANC visit (four and above visit) were had a better experience of adhering to the recommended IFA supplementation. The finding was supported with studies done New Zealand [59], Malawi [58], India [60], Niger [61], Bangladesh [64], and Tanzania [25]. The possible reason for this might be due to the fact that women with registered earlier for ANC visit and who had more frequent visit have an earlier exposure of information which could be more likely to be knowledgeable about the supplement and anemia [50], subsequently end-up with the adherence of the IFA supplementation.

Moreover, this systematic review and meta-analysis found that the commonest barriers of IFA supplementations were fear of side effects and forgetfulness of IFA supplements. This is might be due to those women who have limited antenatal care visit are unaware of the perceived benefits of antenatal IFA supplements and side effects such as heartburn and morning sickness might be consumed a less diverse diet which might less likely to adhere to more IFA supplement according to the recommendation. This is supported by studies done in Iran [69] and Pakistan [70], forgetfulness and absence of family support are the common barriers of adherence. This is also revealed that those who consumed less diversified diet increased the risk of metabolic disturbance for pregnant women [71, 72] and might be reducing the adherence of the supplements [73, 74]. Beyond this, limited family and husbands’ support in sharing of the burden of food or household asset and other healthcare-related events in our setup might increase individuals stress and anxiety [75], women easily forgets to take the tablet, subsequently result in decrease their adherence of IFA supplementation [76, 77].

The results of this systematic review and meta-analysis should be interpreted based on some limitation. The highest heterogeneity of results among studies may be explained by heterogeneity in the characteristics of the studies, setting, and this may have led to insufficient statistical power to detect statistically significant association. Thus, a meta-regression analysis revealed that there was no variation due to sample size and publication year. The studies were conducted only on the five regions which reduce its representativeness for the country and all included studies were cross-sectional which unable show causal relationship and seasonal variation of adherence of the supplementation. Besides, most studies were facility-based which might not represent for those who haven’t ANC visit and some studies have a small sample size, which might affect the estimation.

## Conclusions

More than four of nine pregnant women have adhered to iron and folic acid supplementation**.** This systematic review and meta-analysis revealed that receiving supplemental counseling, knowledge of the supplement; early registration and frequent ANC visit were significantly associated with adherence of the iron and folic acid supplementation. Therefore, provision of strengthened supplemental counselling service, antenatal care services, and improving the knowledge of the supplementation are crucial strategies to increase adherence among pregnant women in Ethiopia. In addition, addressing the barriers of adherence mainly managing of side effects and reducing of forgetfulness to take the tablet through getting family support is mandatory.

## Data Availability

Data will be available from the corresponding author upon reasonable request.

## Appendix

**Table 4:** Adherence of iron and folic acid supplementations and determinants in Ethiopia: systematic review and Meta-analysis

Component	Search terms
#1	("Pregnancy"[Mesh] OR "antenatal Care"[Mesh] OR prenatal care [Mesh} OR "Pregnant Women"[Mesh] OR pregnan*[tiab] OR antenat*[tiab] OR prenat*[tiab])
#2	( Adherence “[Mesh] OR Compliance"[Mesh] OR complain*[tiab] OR adher*[tiab] OR utilization*[tiab] OR utilization*[tiab] OR coverage[tiab]
#3	("iron folic Acid"[Mesh] OR iron[tiab] OR folic acid[tiab] OR "iron folate"[tiab] OR "iron folic acid"[tiab] OR "iron-folic acid"[tiab])
#4	Ethiopia [Mesh] OR Ethiopia [tiab]
#5	((#1 AND #2 AND #3)) AND #4)

## Abbreviations

ANC: Antenatal Care
DALYs: Disability Adjusted Life years
DHS: Demographic and Health Survey
IFA: Iron and folic Acid
LBW: Low Birth Weight
POR: Pooled Odds Ratio
WHO: World Health Organization
